# Role of hippocampal NF-κB and GluN2B in the memory acquisition impairment of experiences gathered prior to cocaine administration in rats

**DOI:** 10.1038/s41598-021-99448-w

**Published:** 2021-10-08

**Authors:** Rosa López-Pedrajas, Inmaculada Almansa, María V. Sánchez-Villarejo, Borja Muriach, Jorge M. Barcia, Francisco J. Romero, María Muriach

**Affiliations:** 1 Departamento de Ciencias Biomédicas, Facultad de Ciencias de la Salud, Universidad Cardenal Herrera-CEU, CEU Universities, Calle Santiago Ramón y Cajal, s/n, 46115 Alfara del Patriarca, Valencia Spain; 2 Departamento de Ciencias Biomédicas, Facultad de Ciencias de la Salud, Universidad Cardenal Herrera-CEU, CEU Universities, Calle Grecia, 31, 12006 Castellón de la Plana, Castellón Spain; 3 Neurobiología y Neurofisiología, Facultad de Medicina, Universidad Católica de Valencia “San Vicente Mártir”, Calle Quevedo 2, 46001 Valencia, Spain; 4 Hospital General de Requena, Paraje Casablanca s/n, 46340 Requena, Valencia Spain; 5 Unitat Predepartamental de Medicina, Universitat Jaume I, Avenida de Vicent Sos Baynat, s/n, 12071 Castellón de la Plana, Castellón Spain

**Keywords:** Mechanisms of disease, Neuroscience, Diseases of the nervous system

## Abstract

Cocaine can induce severe neurobehavioral changes, among others, the ones involved in learning and memory processes. It is known that during drug consumption, cocaine-associated memory and learning processes take place. However, much less is known about the effects of this drug upon the mechanisms involved in forgetting.The present report focuses on the mechanisms by which cocaine affects memory consolidation of experiences acquired prior to drug administration. We also study the involvement of hippocampus in these processes, with special interest on the role of Nuclear factor kappa B (NF-κB), *N*-methyl-D-aspartate glutamate receptor 2B (GluN2B), and their relationship with other proteins, such as cyclic AMP response element binding protein (CREB). For this purpose, we developed a rat experimental model of chronic cocaine administration in which spatial memory and the expression or activity of several proteins in the hippocampus were assessed after 36 days of drug administration. We report an impairment in memory acquisition of experiences gathered prior to cocaine administration, associated to an increase in GluN2B expression in the hippocampus. We also demonstrate a decrease in NF-κB activity, as well as in the expression of the active form of CREB, confirming the role of these transcription factors in the cocaine-induced memory impairment.

## Introduction

Cocaine abuse induces severe neurobehavioral changes that modify neuronal circuits, among others, the ones involved in learning and memory processes. Although several reports have shown an enhancement of conditioned learning and memory processes associated to cocaine consumption at low doses^1,2^, high doses of cocaine may impair spatial memory^3^. We have previously reported that the learning of new tasks was enhanced under cocaine effect in rats. Contrarily, memory consolidation of experiences acquired before twenty days of cocaine administration was impaired and associated to a deficit in NF-κB activity in the frontal cortex of these rats^4^. Other authors have demonstrated the involvement of this transcription factor in synaptic function, neurotransmission, neuroprotection, as well as in learning and memory processes^5^. Interestingly, the activity of NF-κB was not altered in the hippocampus of these rats^4^.

Given these previous results, the purpose of this study was to elucidate if long term exposure to cocaine (36 days) finally diminishes NF-κB activity in the hippocampus of cocaine treated rats, considering that this structure is one of the most important brain areas involved in memory formation^6^ and sensitive to drug abuse effects^7–10^. Furthermore, we aimed to deep into the transcriptional cascades accounting for the memory impairment of experiences acquired prior to drug administration.

In this regard, among the neuroadaptations triggered by cocaine, we find dopamine (DA) hypoactivity^11–13^ and a significantly lower dopamine D2 receptor (D2R) binding after chronic consumption^11,12,14^. Since D2R activation enhances NF-κB transcriptional activity^15,16^, the modulation of its function by cocaine could elicit NF-κB activity impairments. Moreover, DA hypoactivity in the hippocampus might contribute to the memory impairment observed previously by our group^4^ as described Gasbarri et al.^17^.

On the other hand, Kaltschmidt et al. reported that loss of neuronal NF-κB impairs spatial long-term memory formation and suggested a transcriptional cascade where NF-κB could control the CREB signaling pathway^18^. This transcription factor also possesses an essential role in long-term memory formation, as well as in the neuroadaptative mechanisms associated to drug addiction^19^.

We must also consider that the two major kinds of hippocampal-based synaptic plasticity are long-term synaptic potentiation (LTP) and long-term synaptic depression (LTD)^20–22^ and both, require NMDA receptors (NMDAR) activation^20^. NMDARs are heteromeric tetramers principally comprised of two NR1 subunits andtwo of the four variants of NR2 subunits (GluN2A-2D) which are thought to play critical roles in many aspects of central nervous system function and dysfunction, from learning and memory to addiction^23,24^. In addition, calcium entry through the above mentioned extra-synaptic NMDARs, activates a general and dominant CREB shut-off pathway^25^.

Finally, the neurotoxic effects of drugs of abuse have been repeatedly linked to oxidative stress^2,7^, cell death and alteration of autophagic processes^26,27^. NF-κB is a nuclear factor sensitive to oxidative stress, which also acts as a regulator of apoptotic and autophagic processes^20,28–30^. Additionally, CREB and GluN2B have been also linked to autophagic pathways^31,32^. Therefore, we also attempt to confirm if these processes might impair the hippocampus and in consequence affect the memory acquisition in our experimental model of chronic cocaine administration. For this purpose, we also measured the antioxidant enzymes glutathione (GSH) and glutathione peroxidase (GPx), the proapoptotic protein caspase 3 and the levels of Beclin 1, Atg 5, Atg 7 and LC3 I/II (specific markers of macroautophagy) in the hippocampus of cocaine treated rats^11,12,14,31,32^.

## Results

### Morris water maze test

**Figure 1:**
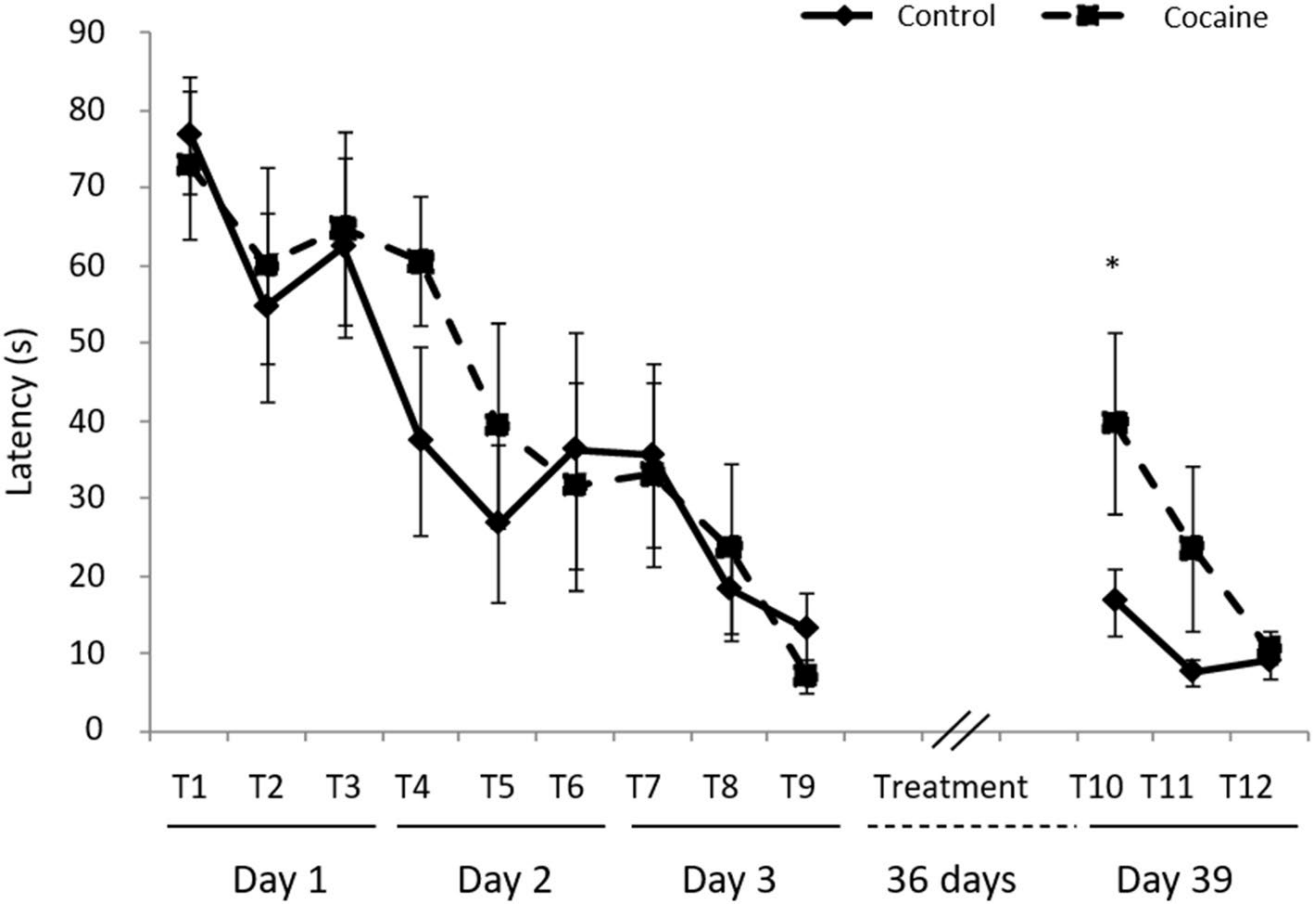
Morris water maze test performance adapted to study memory retrieval in animals from all groups. *p < 0.05 vs. cocaine group trial 9 (n = 8).

Memory retrieval is impaired in cocaine treated animals after 36 days of cocaine administration. A statistically significant increase in the latency (time needed to find the hidden platform) is observed on trial number 10, the first trial after cocaine administration, when compared to latency on trial number 9, the last one before the treatment (p = 0.017, Mann–Whitney Test) (Fig. 1).

No differences were found in the number of times that animals entered in the quadrant were the platform was located, suggesting that locomotor activity is not influencing this task performance (control: 0.17 ± 0.02 and cocaine: 0.13 ± 0.02).

### GluN2B and GluN2A

**Figure 2:**
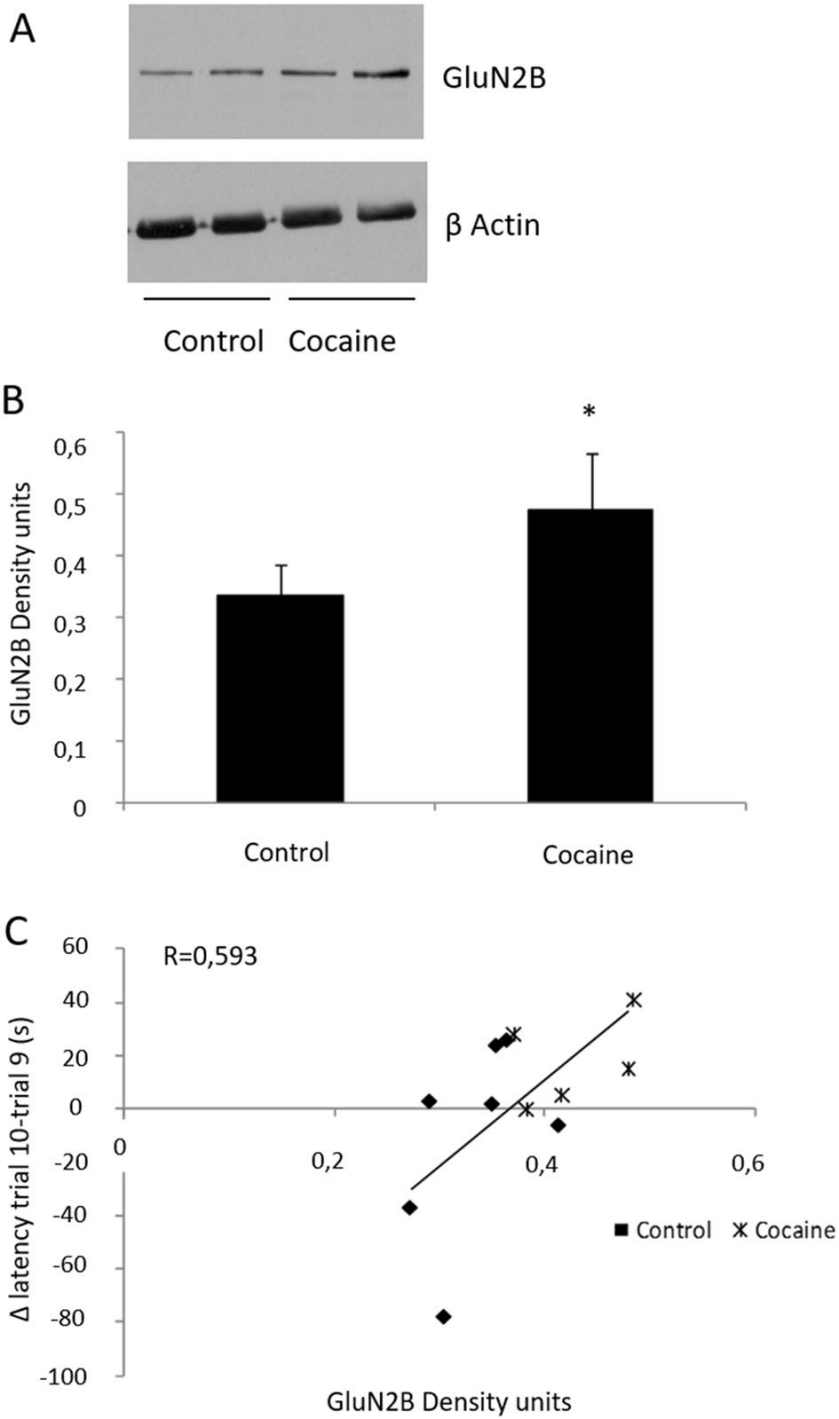
(**A**) GluN2B (180 kDa) and β Actin (42 kDa) western blot in hippocampus. (**B**) Representation of GluN2B density units in hippocampus. *p < 0.05 versus control group (n = 7). Full-length blots are presented in Supplementary Figure 1. Samples derive from the same experiment and that gels/blots were processed in parallel. (**C**) Significant correlation between GluN2B (density units) in the hippocampus and the difference in the latency (seconds) from trial 10- trial 9 (n = 7/6).

GluN2B expression is increased in the hippocampus of cocaine treated rats after 36 days of drug administration when compared to control group (F = 2.209, DF = 12, p = 0.005) (*t*-Student) (Fig. 2A,B). Moreover, there is a statistically significant positive correlation between GluN2B expression in this area and the increase in the latency between trials 9 and 10 (p < 0.05; R = 0.593, F = 5.415, DF = 1) (Fig. 2C). No differences were observed in GluN2A expression in the hippocampus between control and cocaine treated rats (Supplementary Figure 5).

### pCREB/CREB

**Figure 3:**
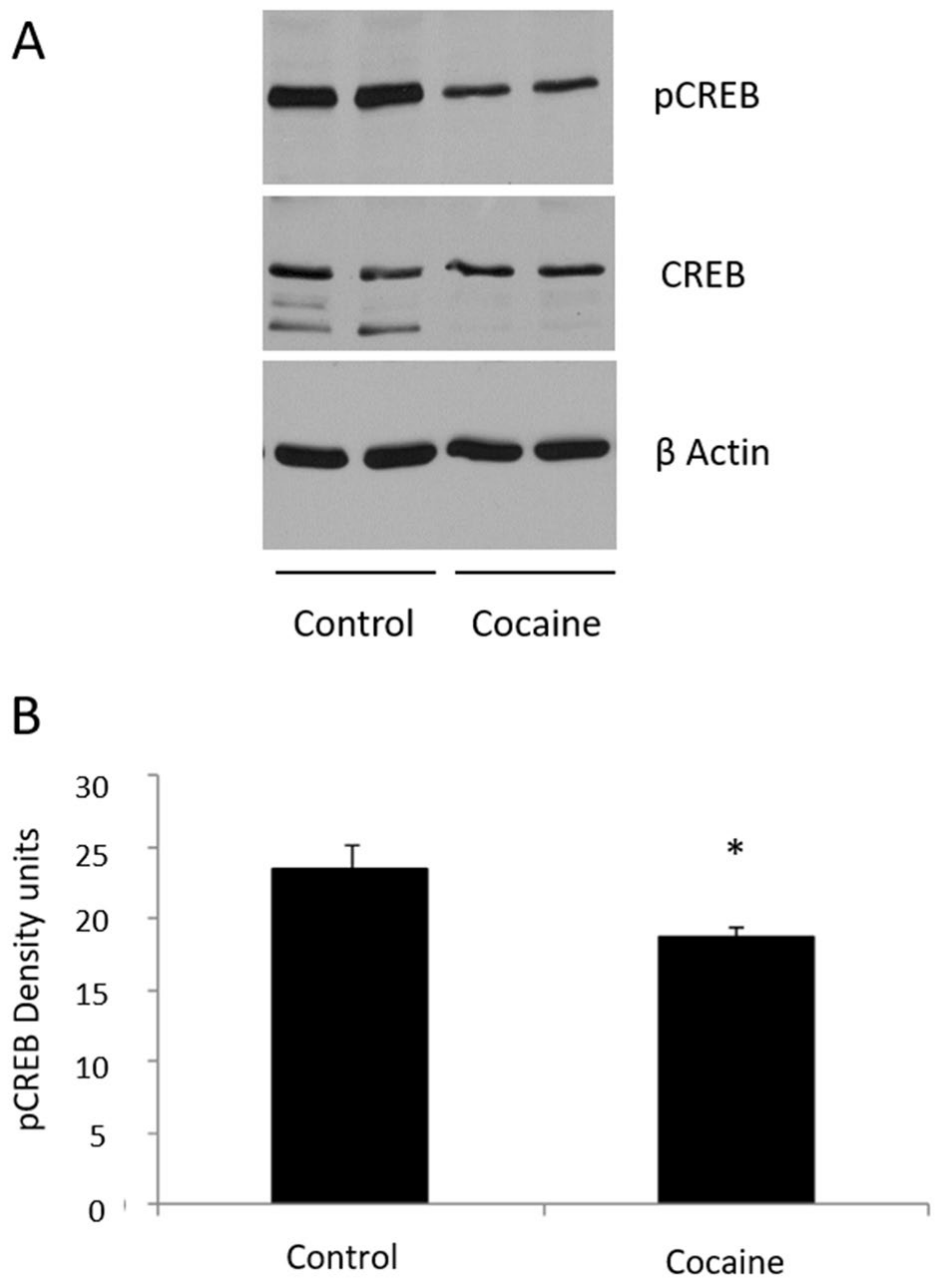
(**A**) pCREB (43 kDa), CREB (43 kDa) and β Actin (42 kDa) western blot in hippocampus (n = 8/6). (**B**) Representation of pCREB density units (pCREB/CREB ratio) in hippocampus. *p < 0.05 versus control group. Full-length blots are presented in Supplementary Figure 2. Samples derive from the same experiment and that gels/blots were processed in parallel.

Figure 3A,B show that the level of pCREB, the active form of CREB, is decreased in the hippocampus of cocaine treated rats (F = 2.576, DF = 12, p = 0.048) (*t*-Student), whereas total CREB level isnot significantly changed after cocaine administration.

### Nuclear factor kappa B activity

**Figure 4:**
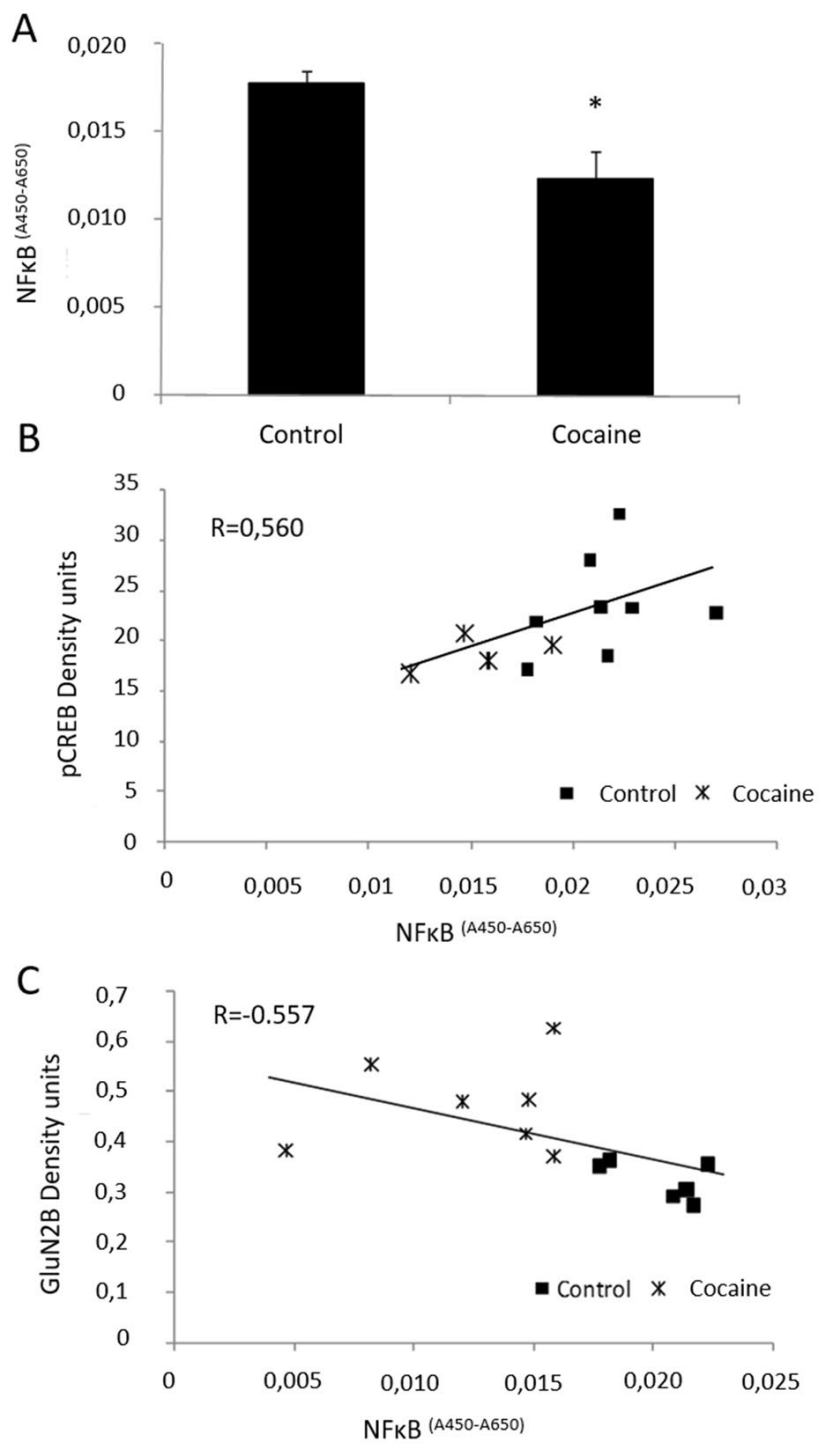
(**A**) NF-κB activity in hippocampus (arbitrary units). *p < 0.05 vs. control group (n = 8/7). (**B**) Significant ccorrelation between NF-κB activity (arbitrary units) and pCREB (density units) in the hippocampus (p < 0.05) (n = 8/5). (**C**) Significant correlation between GluN2B (density units) in the hippocampus and the NF-κB activity (n = 6 / 7).

After 36 days of cocaine administration, NF-κB activity was decreased in the hippocampus (Fig. 4A) when compared to the control group (F = 4.734, DF = 12, p = 0.001) (*t*-Student). Astatistically significant positive correlation between NF-κB activity and pCREB levels is found in this area (p = 0.046; R = 0.56, F = 5.033, DF = 1) (Fig. 4B). NF-κB activity also negatively significantly correlates with GluN2B expression in the hippocampus (p < 0.05; R = -0.557; F = 4.941; DF = 1) (Fig. 4C).

### Dopamine and dopamine 2 receptor

DA levels (control: 1.18 ± 0.10 ng/ml and cocaine: 1.14 ± 0.12 ng/ml) (n = 6/7) and D2DR expression were measure in the hippocampus by ELISA and western blot respectively. No significant differences were found between the control animals and cocaine treated animals in these parameters (Fig. 6).

### Antioxidant defences

GSH concentration (control: 19.99 ± 3.14 nmol/mg prot and cocaine: 21.33 ± 1.86 nmol/mg prot) (n = 8/6) and GPx activity (control: 15.88 ± 3.02 nmol/mg prot × min and cocaine: 16.87 ± 3.88 nmol/mg prot × min) (n = 7/6) in the hippocampus were not affected after 36 days of cocaine administration.

### Caspase 3 activation

In order to measure the possible induction of apoptosis after cocaine administration in the hippocampus, the levels of the active caspase 3 protein and the precursor protein (procaspase 3) were measured. No activation of Caspase 3 was found after cocaine administration and no differences were found in the level of procaspase 3 (Fig. 6).

### Autophagy

**Figure 5:**
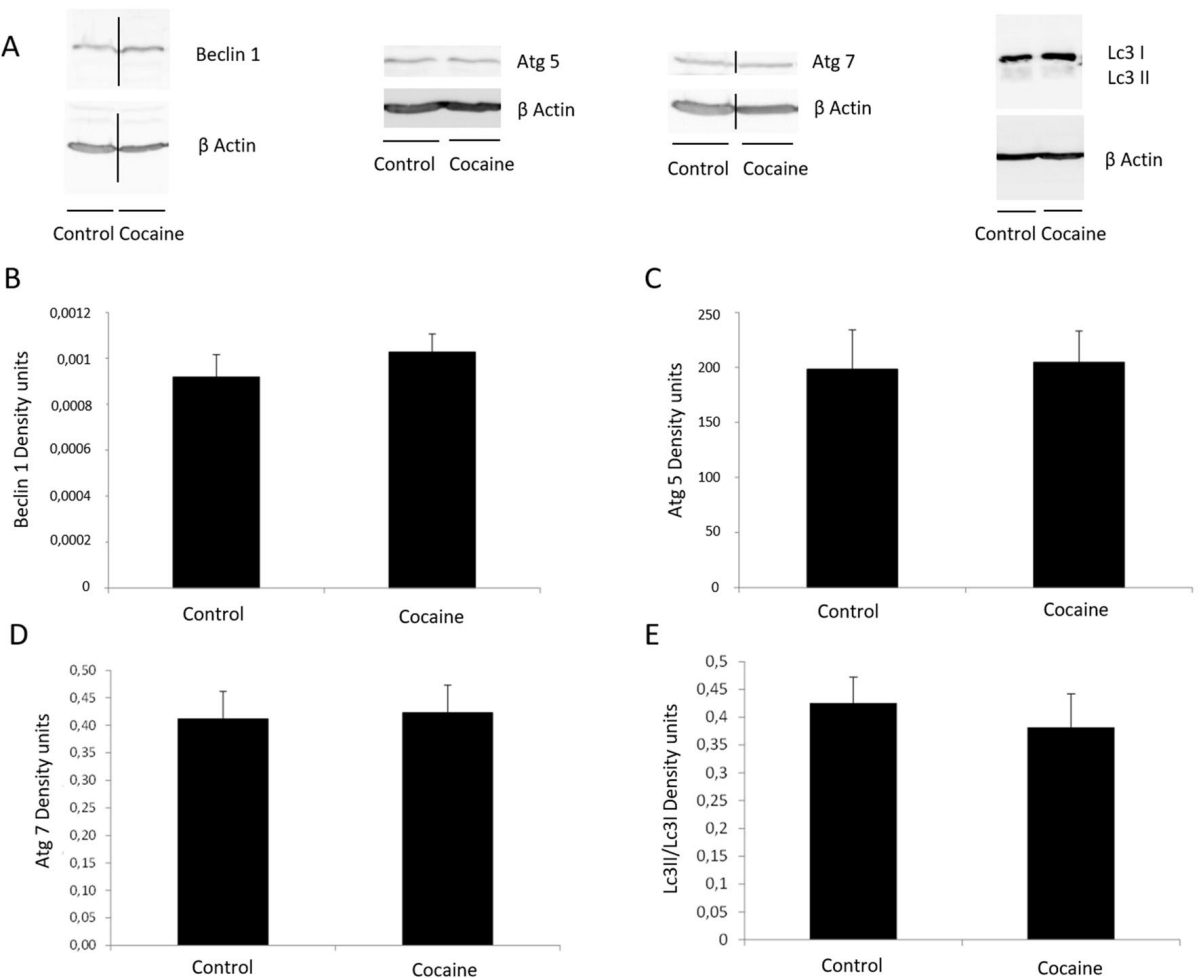
(**A**) Beclin 1 (60 kDa), Atg 5 (56 kDa), Atg 7 (70 kDa), Lc3 I/Lc3 II (16 kDa) and β Actin (42 kDa) western blot in hippocampus. (**B**) Representation of Beclin 1 density units in hippocampus. (**C**) Representation of Atg 5 density units in hippocampus. (**D**) Representation of Atg 7 density units in hippocampus. (**E**) Representation of Lc3 I/Lc3 II density units in hippocampus (n = 7). Full-length blots are presented in Supplementary Figure 3 Samples derive from the same experiment and that gels/blots were processed in parallel.

During autophagy, the cytosolic form of LC3 (LC3I) is conjugated and forms the LC3II, which is considered a specific marker of autophagy. Beclin-1 is one of the most important signaling proteins in autophagosome formation. Atg5 and Atg7 have also been reported to regulate autophagy (28). The levels of Beclin 1, Atg 5, Atg 7 and LC3 I/II were studied in the hippocampus after 36 of cocaine administration, and there were no differences in the expression of these parameters when compared to control group, in thisanimal model of cocaine administration (Fig. 5).

## Discussion

Our results show an increase in the expression of GluN2B subunit (Fig. 2A,B). This result is in accordance with data reported by Barr et al., who described a decrease in the GluN2A/GluN2B ratio in the hippocampus after 7 days of cocaine administration^33^. Moreover, it has been previously demonstrated that while the activation of NMDA receptors containing GluN2A leads to LTP formation, the activation of NMDA receptors containing GluN2B induces LTD^34^. The maintenance of the proper balance between LTP and LTD is necessary for normal cognitive processes. Furthermore, the persistence of LTD may allow acquisition of new information because could weaken previous memory traces, thereby preventing those traces from interfering with newly encoded information^35,36^. However, whereas the “remembering” aspect of memory has been well studied, the mechanisms involved in forgetting are far less explored^37^.

It is hard to discriminate whether cocaine interfered with memory storage and consolidation or induced an impairment in the recall memory network. Considering that cocaine administration started just after the last maze trial, the effects of cocaine could interfere with memory consolidation processes. According to Treves and Rolls^38^ memories are initially stored in the hippocampus and thereafter can be recalled to the neocortex. Likewise, Villarreal et al. have reported an enhancement of spatial memory retention after administration of NMDAR antagonist^39^. In this sense, it is noteworthy that we report an increase in GluN2B expression (previously associated to LTD by Liu et al.^34^), that correlates with an impairment of memories acquired prior to cocaine administration (Fig. 2C). However, no changes were observed in GluN2A expression between control and cocaine treated rats (Supplementary Figure 5). It seems therefore plausible, that cocaine may interfere with hippocampal activity impairing finally, spatial memory storage. Supporting this possibility is the little storage capacity of hippocampus for this type of episodic memories ranging from days to weeks^38^. However, further experiments are necessary to ascertain if GluN2B expression increases earlier after cocaine administration, thus confirming its role in the memory impairment reported (Fig. 1).

Moreover, it has been demonstrated that calcium entry through extra-synaptic NMDA receptors activates a general and dominant CREB shut-off pathway^25^, and there is good electrophysiological evidence that extra-synaptic NMDARs are predominantly composed of GluN2B-containing heteromers^40,41^. Other authors have also suggested a connection between GluN2B and pCREB^42,43^. Our results would agree with this mechanism since we also report a decrease in pCREB expression (Fig. 3A,B). Additionally, CREB is involved in spatial memory formation and long-term synaptic plasticity^44,45^. Thus, the decrease of its expression could therefore be contributing to the impairment of memory retrieval reported (Fig. 1).

**Figure 6:**
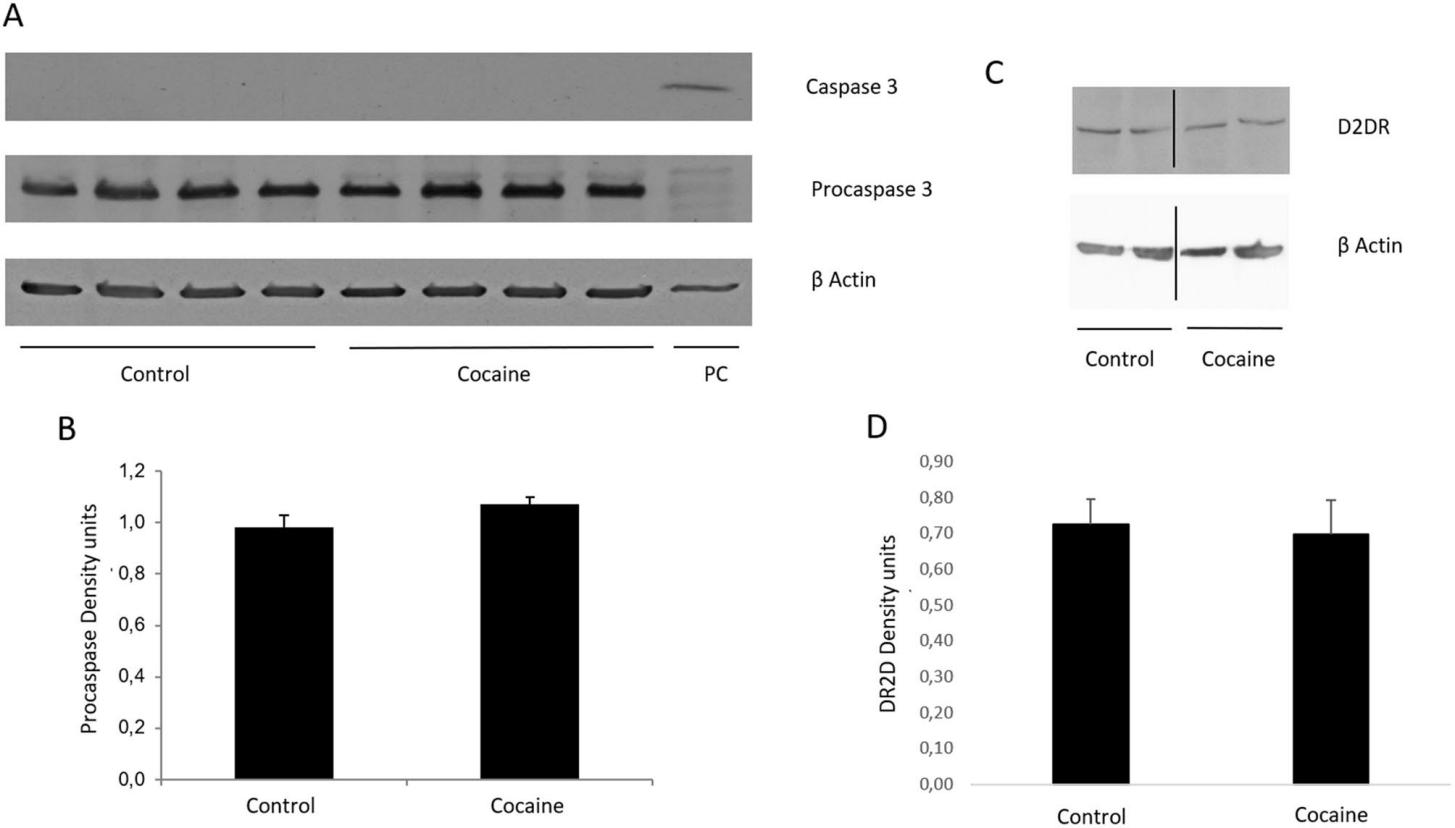
(**A**) Procaspase 3 (35 kDa), Caspase 3 (17/19 kDa), and β Actin (42 kDa) western blot in hippocampus. (**B**) Representation of Procaspase 3 density units in hippocampus (n = 4). (**C**) D2DR (48/51 kDa) western blot in hippocampus. Representation of D2DR density units in hippocampus (n = 7). Full-length blots are presented in Supplementary Figure 4 Samples derive from the same experiment and that gels/blots were processed in parallel.

On the other hand, cocaine repeatedly administered seems to induce DA hypoactivity both, in human addicts and in animal models of cocaine self-administration^11–13^, in association to learning and working memory impairments^17,46^. DA controls the maintenance of long-term memory storage involving DA receptors^47,48^. Therefore, the memory impairment observed in our research could be mediated, at least partially, by DA depletion. Nevertheless, in our experimental model no differences were found in the DA levels after cocaine administration (Fig. 6). This fact is reinforced by the absence of differences in D2DR levels between controls and cocaine treated rats (Fig. 6). These discrepancies could be due to the drug administration model used in this study, since the decrease of DA has been reported in cocaine self-administration models and in our research, cocaine was administered in a controlled manner. However, having that nor DA levels, neither D2DR expression were altered, other mechanisms must be contributing to the memory impairment reported in our model (Fig. 1).

In this regard, we also demonstrate a decrease of NF-κB activity in rat hippocampus after 36 days of cocaine administration (Fig. 4A). We previously described that the activity of this nuclear factor decreases in the rat frontal cortex but not in the hippocampus after 20 days of cocaine exposure^4^. As expected, the results presented herein show that prolonged cocaine exposure finally also promotes a decrease in NF-κB activity in the hippocampus. Snow et al., have previously showed an increase of the NF-κB p65 subunit in hippocampus after training in a spatial memory task^49^. In contrast, the lack of NF-κB is known to impair different kind of memory processes^18,50,51^. Therefore, the spatial memory impairment observed in our study could be mediated at least in part, by the decrease of hippocampal NF-κB activity observed. Having that all the molecular parameters studied in this work are involved physiologically in memory acquisition, the retrieval test performed at the end of the experiments could per se induce transient modifications on these parameters. Further studies including two more groups without a memory recall test, would help to discriminate the effect of this task into the expression or activity of the proteins measured.

On the other hand, Kaltschmidt et al. have previously suggested a transcriptional cascade where NF-κB could control CREB signaling pathway^18^. This connection between NF-κB and pCREB is supported in our model by the statistically significant positive correlation found between both transcription factors in hippocampus (Fig. 4B).

It is noteworthy that we also demonstrate a statistically significant negative correlation between NF-κB activity and GluN2B expression (Fig. 4C). This correlation suggests that NF-κB might influence the effects mediated by NMDARs containing GluN2B, among them the induction of LTD. However, Tai et al. previously suggested that NF-κB could induce GluN2B expression, although they were not able to find any NF-κB-binding site in the promoter of GluN2B^52^. More recently, Xiao et al., have reported in a model of chronic morphine administration, that the administration of an NF-κB activity inhibitor in the dorsal spine is able to abolish the morphine-induced upregulation of GluN2B^53^. The results presented herein, shed some light on the mechanisms that might connect NF-κB activity with the expression of GluN2B and might suggest a dual modulation depending of the experimental model used. Again, further experiments are need to deep in the possible link between these proteins.

Finally, considering the neurotoxic effects of cocaine in terms of oxidative stress, apoptosis and autophagy, we wanted to rule out whether the changes observed in NF-κB activity and CREB and GluN2B expression in rat hippocampus could alter these mechanisms^54,55^ and disturb memory acquisition. Thus, although some reports have related the oxidative stress induced by cocaine with behavioral changes^56^, in our study the antioxidant defenses were unchanged after 36 days of cocaine administration (see results section). Interestingly, we have previously reported the decrease of GSH content and GPx activity in the hippocampus after 20 days of cocaine administration^4^. Since oxidative stress is known to activate NF-κB^54,56^, the normalization of antioxidant defenses after 36 days of cocaine administration could be justified, at least in part, by the decrease of NF-κB activity observed in the hippocampus (Fig. 4A) when compared to the results obtained after 20 days of cocaine administration in which NF-κB remained unaltered^4^.

Moreover, it is widely reported that NF-κB also controls the expression of several pro-survival genes and has been shown to be important in determining neuronal survival/death^57^, as well as it happens with CREB^58^. In addition, Hardingham et al. have reported that while synaptic NMDA receptors have anti-apoptotic activity, stimulation of extra-synaptic NMDA receptors causes loss of mitochondrial membrane potential (an early marker for glutamate-induced neuronal damage) and cell death^25^. It is remarkable that we were not able to detect caspase 3 activation after 36 days of cocaine administration (Fig. 6). This result agrees with other studies that did not observe apoptotic processes in the hippocampus after similar periods of cocaine administration^59–61^.

On the other hand, different in vitro and in vivo data indicate that cocaine promote macroautophagy in other locations of the nervous tissue such as striatum^26–30,62^, but it seems plausible that this autophagic response could be related to inflammatory responses. GluN2B is specifically related to beclin-1, as raft domain, and beclin-1 can be released under GluN2B overdrive as occurring in traumatic brain injury^31^. The lack of autophagic-related alterations herein, is consistent with the absence of hippocampal cell death, toxicity and oxidative disbalance found after chronic cocaine treatment.

Moreover, NF-κB signaling and autophagy are reciprocally modulated^28^^–3^. Thus, Niso-Santano et al., have demonstrated a direct interaction between Beclin-1 and the activation of NF-κB, through IκB kinase^63^. Moreover, Lin et al. have explained a direct binding of p65 to the Beclin 1 promotor^64^. However, the decrease in NF-κB activity (Fig. 4A) was not accompanied by changes in the expression of Beclin 1 and other autophagy related proteins (Fig. 5) in our experimental model.

In conclusion, altogether the results presented herein provide evidence for the possible mechanistic role of hippocampal NMDA receptors (GluN2B), CREB and NF-κB on the observed memory impairment of experiences acquired prior to cocaine administration.

## Methods

### Experimental design

Male Wistar rats weighing 300 g (Charles River Laboratories SA, Barcelona, Spain, RGD Cat# 13,508,588, RRID:RGD_13508588) were used for the experiment. All animal manipulations were done according to the Spanish Law (RD 53/2013) and the experimentaldesignwas approved by Ethics Committee and Animal Welfare of the University CEU Cardenal Herrera (number11/022). The study was carried out in compliance with the ARRIVE guidelines. Rats were individually caged and maintained in a 12 h/12 h light/dark cycle with controlled temperature (20–25 °C) and relative humidity (60%) and had access to food and water ad libitum.

Animals were separated in two groups (n = 8 each one): control and cocaine. Cocaine (Sigma Aldrich) (15 mg/Kg) was administered daily by intraperitoneal injection for 36 days. Control animals received the same injected volume of saline (0.9%).

On the last day, after behavioural tests, rats were injected withpentobarbital and sacrificed by cervical dislocation. Brains were removed, and hippocampus was dissected and homogenized.

Two types of homogenate were made, one for the biochemical analysis and the other for the rest of the assays. For GPx activity and GSH concentration measures the samples were homogenized with 0.1 M PB buffer pH 7,4. Samples for western blot analysis and NF-κB activity were homogenized with lysis buffer (1% triton X-100, 50 mM Tris–HCl pH 8, 150 mM NaCl), supplemented with 1 mM DTT, 10 mM NaF, 1 mM Na2VO4 and 1 × Complete mini protease inhibitor (Roche).When the hippocampi were fully homogenized, they were incubated for 30 min at 4 °C, finally, they were centrifuged for 20 min at 13,000 rpm, keeping the supernatant.

Protein content was measured by the Lowry method^65^ to allow expression of the biochemical results, taken into account the protein content of each sample. Moreover, Bradford method was used to determine protein levels in the other techniques performed^66^.

### Morris water maze test

Spatial learning and memory retrieval were tested using a variant of the Morris water maze test^4,67^, briefly described as follows.

#### Training phase

Two days before starting drug administration, rats were trained to find a hidden platform (10 cm diameter) located 4 cm below the water surface in a circular swimming pool. Three trials per day for three days were performed. Each trial lasted 90 s and the starting point was randomly different between trials. Animals that did not find the platform within this time were guided to the platform by the researcher. Rats remained on the platform for 15 s and then were caged again until the next trial (the inter-trial interval was 45 min). The pool was placed in a room with visual cues. Moreover, rats were habituated prior to each trial, for this purpose the rat was placed in an individual cage for 30 min in the room where the pool was. After the training phase, animals were randomly assigned to the control or cocaine groups.

#### Test phase

Memory retrieval was tested on the last day of experiment with 3 additional trials.

Latency time to find the hidden platform was measured in seconds, as well as the number of entrances on the quadrant where the platform was located.

### Nuclear factor kappa B activity

NF-κB is in a latent form in the cytoplasm bound by the IκB, NF-κB inhibitory protein. NF-κB-inducing stimuli activate the IκB kinase complex that phosphorylates IκB. IκB degradation exposes the DNA-binding domain and nuclear localization sequence of NF-κB and permits its stable translocation to the nucleus and the regulation of target genes. TransAM NF-κB p65 (Active Motif, Rixensart, Belgium) is an ELISA-based kit to detect and quantify this transcription factor subunit activation. Both positive and negative controls were assayed together with the samples. Optical density was measured at 450 nm and a reference wavelength at 650 nm in a multilabel counter (ELISA Plates Spectrophotometer, Multiskan Ascent, Labsystem). Results are represented as arbitrary units.

### Western blot analysis

The homogenized samples were run on 10–12% sodium dodecyl sulfate (SDS) (Sigma Aldrich, Spain) polyacrylamide gels and transferred to nitrocellulose membranes (Amersham Biosciences, UK), which were blocked in 5% skim milk in Tris Buffered Saline (TBS) (Sigma Aldrich, Spain) and 0.1% Tween 20 (Sigma Aldrich, Spain), for 1 h. Thereafter, samples were incubated with the primary antibody overnight at 4 °C. Primary antibodies against pCREB (Millipore Cat# 06-519, RRID:AB_310153, Darmstadt, Germany) and CREB (Santa Cruz Biotechnology Cat# sc-186, RRID:AB_2086021, Santa Cruz, California), GluN2B (Millipore Cat# AB1557P, RRID:AB_11214394, Darmstadt, Germany), GluN2A (Abcam, Cat# Ab124913), (procaspase 3 (Santa Cruz Biotechnology Cat# sc-7148, RRID:AB_637828, Santa Cruz, California), caspase 3 (Cell Signaling Technology Cat# 9664, RRID:AB_2070042, Leiden, The Netherlands), D2R (Santa Cruz Biotechnology Cat# sc-5303, RRID:AB_668816, Santa Cruz, California), Beclin 1 (Santa Cruz Biotechnology Cat# sc-11427, RRID:AB_2064465, Santa Cruz, California), Atg-5 (Novus Cat# NB110-53818, RRID:AB_82858, Abingdon, United Kingdom), Atg-7 (Cell Signaling Technology Cat# 2631, RRID:AB_2227783, Leiden, The Netherlands), Lc3 (Cell Signaling Technology Cat# 2775, RRID:AB_915950, Leiden, The Netherlands) and peroxidase β-Actin (Sigma-Aldrich Cat# A3854, RRID:AB_262011, Spain), were used. Bound antibody was visualized using horseradish peroxidase-coupled secondary anti-rabbit (Santa Cruz Biotechnology Cat# sc-3837, RRID:AB_650507,Santa Cruz, California) and peroxidase-coupled secondary anti-mouse (Thermo Fisher Scientific Cat# 31437, RRID:AB_228295, Spain), then the membrane was incubated 1 h at room temperature. Finally, the signal was detected with enhanced chemiluminescence (ECL) developing kit (Amersham Biosciences, UK). Blots were quantified by densitometry using Quantity One software (Quantity One® Version 4.6.3. https://www.bio-rad.com/es-es/product/quantity-one-1-d-analysis-software?ID=1de9eb3a-1eb5-4edb-82d2-68b91bf360fb ) or ImageJ Version 1.25. (https://imagej.net/software/fiji/).

### Dopamine research assay

Dopamine (DA) secretion was measured using an enzyme immunoassay for the quantitative determination of DA (ELISA) (LDN, Nordhorn, Germany) according to the manufacturer´s instructions. Hippocampus homogenates were collected and immediately used for DA quantitation through ELISA according to the instructions. Optical density was measured at 450 nm and a reference wavelength at 650 nm in a multilabel counter (ELISA Plates Spectrophotometer, Multiskan Ascent, Labsystem).

### Biochemical assays: antioxidants defenses

#### GSH concentration

The samples used to find the concentration of GSH was acidified with 20% of perchloric acid (Panreac, Spain). GSH content was quantified following the method of Reed et al. (1980), based on the reaction of iodacetic acid with the thiol groups followed by a chromophore derivatization of the amino groups with Sanger reactant (1-fluoro-2,4-dinitrobencene) (Sigma Aldrich, Spain), giving rise to derivatives which are quickly separated by means of HPLC, thus allowing a quantification of nanomolar concentrations of GSH^68^.

#### GPx activity

GPx, which catalyzes the oxidation by H_2_O_2_ of GSH to its disulfide GSSG, was assayed spectrophotometrically as reported by Lawrence et al. (1978)^69^ by monitoring the oxidation of NADPH at 340 nm. The reaction mixture consisted of 240 mU/mL of glutathione disulfide reductase (Sigma Aldrich, Spain), 1 mM GSH (Sigma Aldrich, Spain), 0.15 mM NADPH (Biotest, Valencia, Spain) in 0.1 M potassium phosphate buffer, pH 7.0, containing 1 mM EDTA (Sigma Aldrich, Spain) and 1 mM sodium azide (Sigma Aldrich, Spain); a 50 μL sample was added to this mixture and allowed to equilibrate at 37 °C for 3 min. The reaction was started by the addition of H_2_O_2_ (Sigma Aldrich, Spain) to adjust the final volume of the assay mixture to 1 mL.

### Statistical analysis

Results are presented as mean values ± SEM. Statistical significance was assessed by Student *t* test or Mann–Whitney Test, previously the data were analysed with the Kolgomorov-Smirnov test and the Levene test. The level of significance was set at p < 0.05. The correlations between the data were done using the linear regression test. The data were analysed with the SPSS program.

## Supplementary Information

Supplementary Information 1.

## Data Availability

The datasets generated during the current study are available from the corresponding author on reasonable request.
