# Supplementary figures and images for: Role of hippocampal NF-κB and GluN2B in the memory acquisition impairment of experiences gathered prior to cocaine administration in rats
Source: Sci Rep. 2021 Oct 8;11:20033. doi: 10.1038/s41598-021-99448-w (PMC8501066; doi:10.1038/s41598-021-99448-w)

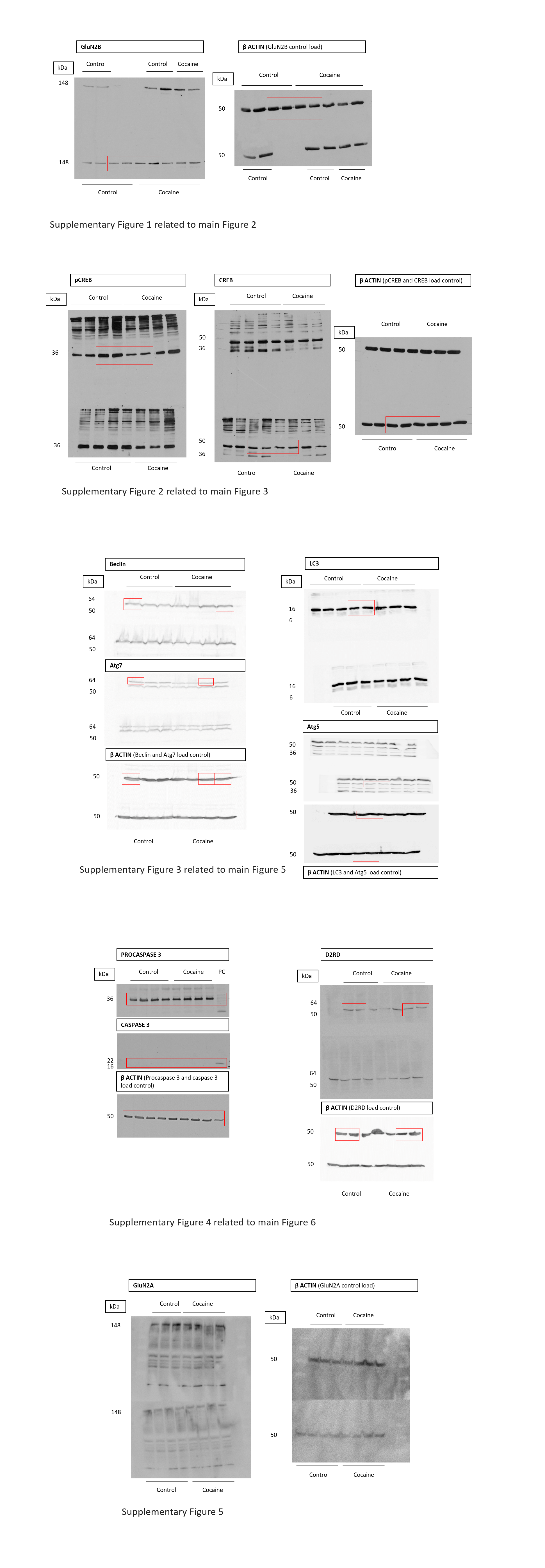

Supplement: Supplementary file 1 — Supplementary Information 1. [file 41598_2021_99448_MOESM1_ESM.tif]
